# 37606 Enhanced radiation therapy using chlorin-e6 conjugated gold nanoparticles

**DOI:** 10.1017/cts.2021.513

**Published:** 2021-03-30

**Authors:** Samir V. Jenkins, Robert J. Griffin

**Affiliations:** University of Arkansas for Medical Sciences

## Abstract

ABSTRACT IMPACT: Improved radiation treatment will yield higher doses at the tumor site, while reducing damage to healthy tissue, which will improve clinical outcomes. OBJECTIVES/GOALS: Development of gold nanoparticles covalently linked to a photosensitizer for use to enhance radiation therapy. The particles will be thoroughly characterized and the mechanism uncovered. The efficacy of these particles will be tested in a murine system. METHODS/STUDY POPULATION: Gold nanoparticles were synthesized and coated with amine-terminated poly(ethylene) glycol then covalently conjugated to chlorin e6, a known, FDA approved photosensitizer. The system was characterized using UV-Vis spectroscopy, transmission electron microscopy, and nanoparticle tracking analysis. The generation of reactive oxygen species following X-irradiation was measured. Enhanced cell killing was measured clonogenically and in vivo efficacy and tumor pathology was assessed in a murine system. Further studies will determine the optimum combination of particle shape, photosensitizer structure, and ratio of components, as well as the optimal dosing schedule. RESULTS/ANTICIPATED RESULTS: Conjugation of the particle to the photosensitizer was successfully achieved, and the molecule was detectable by UV-Vis spectroscopy. TEM and NTA showed no aggregation of the particles, and an increase in reactive oxygen species generation was observed. The conjugates significantly increased cell killing during radiation treatment, while neither the particle alone or the photosensitizer significantly affected clonogenic survival at the same concentrations. Pathology of breast tumors grown in immunocompetent mice showed a significant increase in necrotic tissue following a single 20 gy treatment when the conjugate was present. DISCUSSION/SIGNIFICANCE OF FINDINGS: Radiation therapy is widely used clinically and it is a highly localized form of treatment. However, the total dose of radiation is limited largely to prevent injury to adjacent normal tissue. This conjugate has the potential to increase the effective dose in the tumor thereby reducing damage to healthy tissue and providing a more effective therapy.

